# Brewer’s Spent Grains—Valuable Beer Industry By-Product

**DOI:** 10.3390/biom10121669

**Published:** 2020-12-13

**Authors:** Mateusz Jackowski, Łukasz Niedźwiecki, Kacper Jagiełło, Oliwia Uchańska, Anna Trusek

**Affiliations:** 1 Department of Micro, Nano and Bioprocess Engineering, Faculty of Chemistry, Wroclaw University of Science and Technology, Norwida 4/6, 50-373 Wroclaw, Poland; 233652@student.pwr.edu.pl; 2 Department of Mechanics, Machines, Devices and Energy Processes, Faculty of Mechanical and Power Engineering, Wroclaw University of Science and Technology, Wybrzeze Wyspianskiego 27, 50-373 Wroclaw, Poland; lukasz.niedzwiecki@pwr.edu.pl; 3 Faculty of Veterinary Medicine, Wroclaw University of Environmental and Life Sciences, Norwida 31, 50-375 Wroclaw, Poland; 110419@student.upwr.edu.pl

**Keywords:** brewer’s spent grains (BSG), waste management, circular economy, brewing by-product, beer, biosorbent, biomethane, solid fuel, nutrient, hydrochars, natural compounds extraction

## Abstract

The brewing sector is a significant part of the global food industry. Breweries produce large quantities of wastes, including wastewater and brewer’s spent grains. Currently, upcycling of food industry by-products is one of the principles of the circular economy. The aim of this review is to present possible ways to utilize common solid by-product from the brewing sector. Brewer’s spent grains (BSG) is a good material for sorption and processing into activated carbon. Another way to utilize spent grains is to use them as a fuel in raw form, after hydrothermal carbonization or as a feedstock for anaerobic digestion. The mentioned by-products may also be utilized in animal and human nutrition. Moreover, BSG is a waste rich in various substances that may be extracted for further utilization. It is likely that, in upcoming years, brewer’s spent grains will not be considered as a by-product, but as a desirable raw material for various branches of industry.

## 1. Introduction

**Figure 1:**
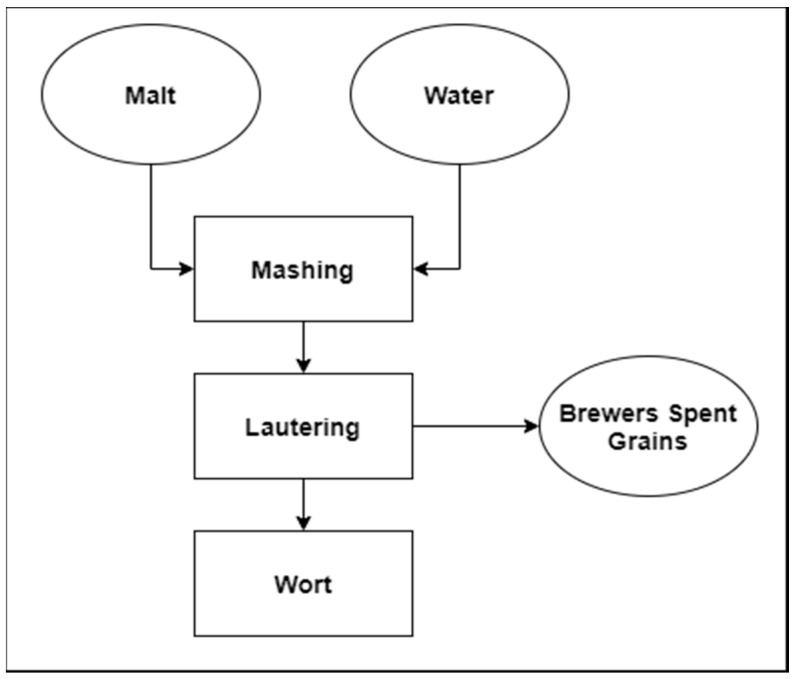
Diagram of beer wort production with an emphasis on the main solid by-product.

Barley and wheat were domesticated during the Neolithic Revolution, about 10,000BC, which changed human behaviour from hunting and gathering into agriculture [1,2,3]. Those grains were used mostly for baking. The first known brewers were the Sumerians, who started brewing in at least 4000BC [4]. Evidence shows that beer was widely known in the ancient world [5,6]. Since then, the beer industry started to grow from small manufactures to the big industrial breweries known today. In modern breweries, there are four main ingredients for beer production; water, malt, yeasts, and hops. Water is a solvent, for sugars extracted from malt during the mashing process, and it creates an environment for yeasts [7]. Sugars, required for fermentation, are delivered into wort by malt [8]. Yeasts are the workhorses of fermentation; thanks to them, the final product receives CO_2_, ethanol and higher alcohols responsible for the aroma profile of finished beer [9]. Hops are responsible for herbal aroma and bitterness of the finished product [10]. Beer production starts with the grinding of malt grains and unmalted materials (if used). Such raw material is mixed with water and heated up to the temperatures optimal for enzyme activity. This phase is named mashing, and its purpose is to extract sugars from grains. The essential enzymes during that stage are alpha and beta amylases present in malt. Beta-amylase cleaves every second a-1,4 bond in starch chains, starting from its non-reducing end. Alpha-amylase cleaves mentioned bonds in random order. Mashing ends with heating the mixture up to 78 °C in order to denature remaining enzymes and stop their activity. The next step is lautering. During that process, the wort is being separated from spent grains (Figure 1). Subsequently, the filtrate is being boiled with hops. The purpose of that step is to sterilize the wort, and to add hop bitterness and aroma. Finally, the wort has to be cooled down, and yeasts are added prior to the fermentation. The main solid by-product in the brewing industry is brewer’s spent grains (BSG) obtained during lautering [9,11].

Industrial-scale breweries produce high quantities of mentioned wastes and are able to deliver it constantly. According to Eurostat, 34 billion L of beer was produced in the European Union in 2019 [12]. That means that large quantities of brewer’s spent grains are produced yearly. Such by-product is rich in cellulose, hemicellulose, lignin, and proteins (Table 1). It may be feasible to use them in the neighbourhood of such factories due to the high costs of transport. The aim of that overview is to show the possible ways to utilize brewer’s spent grains.

## 2. Activated Carbon Production and Sorption Properties

Activated carbon is used today for many different applications, such as decolourising, solvent recovery, water treatment, deodorizing, treatment of different gases for removal of impurities, precious metals recovery and catalysis [28]. The process of production of activated carbon consists of two principal processing steps: carbonization of biomass and activation [29]. Typically, two distinct types of activation are used for developing of a highly porous structure, i.e., thermal-physical or chemical activation [28,30,31]. Firstly, initial treatment is done to produce the substrate for activation by carbonization at 400–500 °C [28]. During thermal activation partial gasification, using a mild oxidizing gas such as CO_2_, steam or flue gas at 800–1000 °C, is performed in order to develop the porosity and surface area [28]. Such treatment has been investigated so far, for many different types of biomass, using various gasification agents [32,33,34,35].

On the other hand, chemical activation is typically performed, using inorganic additives, such as acids or bases, before carbonization [28,36,37,38,39]. These additives degrade and dehydrate the cellulosic materials during carbonization at 250–650 °C [28]. Obtained modified biochar (BC), depending on used oxidizing gases or acidic or basic oxidizing solutions, contains different chemical functional groups (e.g., phenolic, carboxylic, carbonylic, etc.), making it a very attractive tool for wastewater treatment, CO_2_ capture, toxic gas adsorption, soil amendment, supercapacitors, catalytic applications, etc.

Research shows that BSG is a very promising material to produce activated carbon in a thermal activation way [3]. Relatively high nitrogen content in a dry mass [40,41,42,43] between 2 and 5 percent (different types of malts could have different nitrogen content in a dry mass) have a positive impact for adsorption properties in produced activated carbon. Additionally, activated carbons from BSG could have similar or even better adsorption properties of ions (e.g., Cr or Fe) and phenolic compounds than commercial activated carbons [41,42,44].

Non-activated carbon materials from brewer’s spent grain could be used in adsorption processes too. Research shows that BSG after saturation of the active sites with hydrogen cations could, with good effectiveness, adsorb the copper [45] or other heavy metals [46] cations from water solutions. Dyes could also be removed by using a BSG as a cheap and eco-friendly biosorption material [47,48]. Moreover, It could be transformed into a magnetic form by a treatment, using perchloric acid stabilized magnetic fluid containing iron oxide nanoparticles [48]. One of the most critical factors in ions and dyes biosorption on BSG is pH. To obtain the maximum efficiency, pH has to be chosen under the specified ion or dye—one of the most important factors in these cases is a structure of adsorbed particles and type of ion (cationic or anionic).

## 3. Biomethane Production

Biowaste material is reported as a good source for biomethane production. Selective collection of biowastes is practised in numerous cities across Europe [49]. The main problem with typical biowastes from urban areas is variable composition and indigestible materials, like plastic [50]. What is more, its collection and transport are costly [49]. Breweries are potential sources of a high amount of standardized biological by-product—brewer’s spent grains. In case of large, industrial breweries, such biowaste is accessible every day. Wang et al. studied biomethane production from BSG. They proposed to start a process with enzymatic pre-treatment in order to break down cellulose, hemicellulose, and proteins into small monomers. Such hydrolysate was anaerobically digested into methane using continuously stirred bioreactor, sequencing batch bioreactor with expanded granular sludge bed. The last of the mentioned methods was the most efficient, with 56% of total organic matter converted into methane [51]. Vitanza et al. reported that the conversion of organic matter in BSG using anaerobic digestion reached 81% [52]. Such a process may be more effective when microwave-assisted alkali pre-treatment is applied [53]. In that case, 46% of lignin and 38% of hemicellulose is being removed. On the other hand, this kind of pre-treatment requires additional costs for electricity and caustic soda [53]. Dudek et al. observed the addition of biochar to anaerobic digestion of BSG. This study showed that 5% addition of biochar increased production of biogas, while the reaction rate did not change [54]. Moreover, the whole process of anaerobic digestion may be improved by the addition of microelements. Studies showed that supplementation with Mg, Co, K and smaller amounts of Ni and Fe increases the stability of methane production [55].

## 4. Thermal Valorization of BSG

### 4.1. BSG as a Solid Fuel

**Figure 2:**
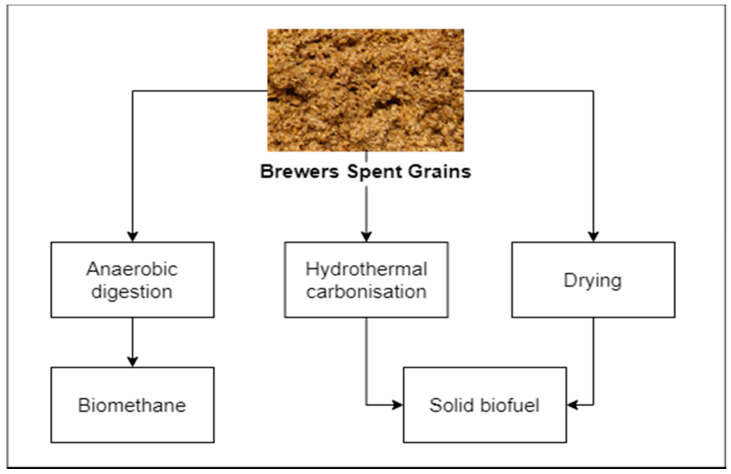
Energetic valorization of BSG.

Basic fuel properties, reported by many researchers, suggest that BSG is promising as a solid fuel (Figure 2) [56,57]. Reported carbon content reported is typically ranging between 45% up to approx. 49% on the dry basis [56,57], which makes BSG not significantly different in terms of its fuel properties, in comparison to lignocellulosic biomass. Additionally, ash content varies between 2 and 6% [57,58,59], which is similar to different types of agricultural biomass [60,61,62,63,64,65,66]. However, high moisture content, exceeding 70% [56,57], is a significant obstacle in the use of BSG as a solid biofuel. Drying is possible but requires energy and bulky installations due to relatively high residence time, e.g., the order of magnitude of 100 min was reported by Arranz et al. [67] needed to obtain moisture reduction of 0.2 of the original value, corresponding with the moisture content of approx. 15%. Moreover, the energy required for the drying process should not be overlooked. Stroem et al. [68] reported drying energy, for superheated steam drying of BSG, ranging between 0.65 and 1.45 MJ/kg of removed water, when latent heat recovery from steam was included in the balance [68].

### 4.2. Hydrothermal Carbonization as a Thermal Valorization Method for Wet Types of Biomass

HTC is a thermal valorization process, typically performed at elevated temperatures (typically 200 to 260 °C) in subcritical water, at elevated pressure [69,70]. The use of such a process can enhance mechanical dewatering, which has already been reported for various wet types of biomass [71,72,73].

The ionic constant of water is significantly increased, and water behaves as a non-polar solvent at 200–280 °C [73,74,75,76,77,78,79]. A multitude of reactions occurring at the same time, with the output of multiple different products, can be considered characteristic for HTC of complex substances such as different types of biomass [70]. The HTC process starts with hydrolysis [69]. This is followed by dehydration and decarboxylation [69,80]. Dehydration decreases the amount of hydroxyl groups (OH) [69]. The decrease in the amount of OH groups also causes a lower O/C ratio. Decarboxylation decreases the amount of carboxyl (COOH) and carbonyl (C=O) groups, also slightly decreasing the O/C ratio of the solid product [69]. This is followed by polymerization and aromatization [69,80]. A decrease in the number of hydroxyl groups is the key aspect in making hydrothermally carbonized biomass more hydrophobic, lowering its equilibrium moisture content [81] and making physical dewatering easier [69]. The ability to decrease O/C ratio is beneficial when valorization is performed, aiming at improving the results of subsequent pyrolysis [82,83,84]. Moreover, the process of hydrothermal carbonization can change the biomass in terms of the composition of the inorganic fraction [70,85]. Furthermore, some studies reported relatively easy pelletizing of hydrochars [86]. This makes hydrothermal carbonization a prospective valorization process for low-quality solid biofuels, especially when wet biomass is concerned as a potential feedstock.

### 4.3. The Effect of Hydrothermal Carbonization of BSG

Slight improvement in mechanical dewatering, thanks to HTC of BSG, was observed by Jackowski et al. [72]. Moreover, the GC-MS analysis of the liquid HTC effluent indicated that it contains organic compounds that could be used to produce biogas in the anaerobic digestion [72]. Similarly, Poerschmann et al. [87] found phenols, benzenediols, and fatty acids in the liquid by-products of HTC of BSG, concluding that the release of such compounds is an effect of the presence of bound lipids in the feedstock [87]. HTC of spent grain from a big scale brewery, performed by Arauzo et al. [88], resulted in an improvement in fuel properties. Higher heating value (HHV) increased, accompanied by a decrease in the ash content, especially for high water: biomass ratios [88]. The study deemed low temperatures of the HTC process especially suitable, thanks to the high content of hemicellulose in the feedstock [88]. Jackowski et al. observed that the yield of HTC can be determined by an indirect method [89]. The study also confirmed that HTC could increase the heating value of BSG and decrease the O/C ratio [89], indicating its suitability as a valorization method suitable for subsequent pyrolysis.

A Py-GC-MS analysis of BSG and corresponding hydrochars were performed by Olszewski et al. [90]. Relatively low pyrolysis temperature for spent grains resulted in a release of a significant amount of *N*-compounds, which was attributed to weakly bonded proteins present in the feedstock [90]. On the other hand, fewer *N*-compounds was released during pyrolysis of hydrochars, owing to the Maillard reactions producing more stable *N*-heterocyclic structures [90]. A single-step and two-step BSG pyrolysis process, consisting of HTC and pyrolysis, was compared by Olszewski et al. [91]. Hydrothermal carbonization, performed at temperatures between 180 and 260 °C, resulted in the removal efficiency of inorganics, ranging from almost 60% to more than 95% for K, approx. 45% to approx. 55% for P, and approx. 35% up to approx. 75% for Na [91]. Moreover, HTC performed at 180 and 220 °C, and pyrolysis at 600 °C resulted in increased BET surface for pyrochars from a two-step process, when comparing to single-step pyrolysis at the same temperature [91]. Jackowski et al. observed that the yield of HTC can be determined by an indirect method [89]. The study also confirmed that HTC could increase the heating value of BSG and decrease the O/C ratio [89].

## 5. Extraction of High-Value Compounds from BSG

Due to the multitude of compounds contained, the brewer’s spent grain undergoes extraction processes to obtain substances with the desired properties. BSG undergoes many different extraction processes, such as alkaline hydrolysis [92], enzymatic hydrolysis [93], microwave-assisted extraction [94], solvent extraction [24], supercritical carbon dioxide extraction [95], ultrasound-assisted extraction [96] etc. The products that can be obtained by extraction are:

### 5.1. Arabinoxylans, Polyphenol, Antioxidants and Glucose

Arabinoxylan is a polysaccharide consisting of two pentose sugars: xylose and arabinose [97]. Among other hemicelluloses, cellulose, and lignin, it is part of the dietary fibre found in BSG. It can bind to polyphenols such as ferulic acid and *p*-cumaic acid. Arabinoxylans can be recovered by ultrasound-assisted extraction [96], microwave-assisted extraction [94] or HCl and ethanol extraction (after previous protein extraction) [98].

Studies show that supercritical extraction of CO_2_ with ethanol 60% *v/v* at 35 MPa, 40 °C at an extraction time of 240 min allows a good recovery of phenolic or flavonoid fractions [95,99]. The extract obtained is characterized by good antioxidant properties. Phenolic fractions can also be obtained by solvent extraction (acetone–water mixture) [24]. Good recovery of ferulic and *p*-coumaric acids is provided by the BSG alkaline extraction [92] and solvent extraction (acetone: water mixture) [24,100].

Alonso-Riaño et al. characterized extraction, and determined kinetics of the water, ultrasound-assisted extraction of polyphenol compounds from BSG [101]. Experimental data were in good agreement with both power law and the Weibull model [101]. Ultrasound-assisted extraction achieved similar productivity, after 30 min of treatment, in comparison to enzyme hydrolysis [101]. Birsan et al. compared conventional maceration, microwave and ultrasound-assisted extraction, using BSG from light and dark beer as well as their mixtures [102]. Microwave and ultrasound extraction did not improve the total polyphenol yield when compared to the conventional maceration method [102]. Tan et al. investigated the use of *Bacillus subtilis WX-17* to improve the nutritional value of BSG in a solid-state fermentation [103]. The total amount of unsaturated fatty acid and the total antioxidant quantity increased by 1.7 and 5.8 times, respectively [103]. Zuorro et al. investigated the extraction of phenolic antioxidants from BSG, using acetone–water and ethanol–water mixtures as extraction solvents [104]. The extraction yield was maximum at 60% (*v*/*v*) organic solvent concentration, for both solvents [104].

### 5.2. Proteins

Due to the high protein content (about 20% in dry matter), BSG is a good potential source of vegetable protein for the food industry. In the case of protein extraction, the selectivity of the extraction process is crucial. Alkaline treatment of BSG, by Du et al. [105], resulted in the extraction yield of 21.4% and purity of 60.2% for proteins extracted from BSG. In case of a combination of alkaline pretreatment with diluted acid, a very high degree of extraction was obtained (even 95%). However, the selectivity of this process was not good enough, because part of lignin and hydrocarbons contained in BSG was dissolved together with proteins [106]. Good selectivity, with lower horizontal extraction (about 65%) was obtained with hydrothermal pretreatment, which significantly required lower temperature and did not require the use of chemicals [106].

Good results of the extraction of proteins from BSG (up to 80%) were achieved with the use of carboxylate salt—urea DES [107]. The disadvantage of this technology is the residual DES in the protein product, but in a case when a substitute for urea will be gained, this method could be attractive for making human nutrition products.

Another promising method is the use of ultrasounds for enzymatic hydrolysis of proteins from BSG [27]. By using ultrasound pretreatment, the efficiency of protein separation is increased (from 61.6 to 69.8%), the time of enzymatic reaction is shortened (by 56%), and the cost of enzyme use can be reduced (even 73%).

## 6. Sustainable Materials

Next interesting application of brewer’s spent grains are construction materials. Nowadays, there are numerous attempts to utilize biological by-products in such a way [108,109,110]. Brewer’s spent grain seem to be feasible for fillers and reinforcement materials. Furthermore, this kind of practice allows reducing costs of biocomposites. Zedler et al. investigated the modification of rubber with BSG and ground tire rubber. Two curing systems were tested; sulfur-based and dicumyl peroxide. Results showed that biocomposites cured with sulfur represent better acoustic and physicomechanical absorption. What is more, such a curing method does not influence the thermal stability of the product [111]. Formela et al. conducted experiments on reinforcing polyurethane foam with brewer’s spent grains and ground tire rubber. Results showed that both waste fillers might be used as cheap and environment-friendly reinforcement phase for polyurethane foam. Moreover, combinations in spent grain and tire rubber ratio allows to design composites with various properties, which enriches the spectrum of their possible applications [112]. Another way to utilize biowastes from breweries is to modify building materials with brewer’s spent grains. Ferraz et al. tested ceramic bricks made with powder mixtures enriched with 5, 10, and 15% addition of dried BSG to brick raw material. Incorporation of 5% of BSG by mass seems to be the best compromise between high mechanical bending strength and low thermal conductivity. For the obtained product, mechanical bending strength reached 15 MPa, and it was 12% less than the strength of the unmodified brick. On the other hand, thermal conductivity decreased by 28% in comparison with unmodified brick and was equal to 0.46 W/mK [113]. Russ et al. conducted a large-scale experiment on BSG enriched bricks. Spent grains were 3.5% of brick raw material. Results showed that BSG might be a substitute for sawdust used in the brick industry. The bricks produced with spent grains represented comparable or even higher strength, increased porosity, and a reduced density after firing in comparison to standard clay bricks. Moreover, the experiment demonstrated that the obtained product met the specifications mentioned in German regulations and may be introduced to the market. Authors report that there were no problems with the production process [114]. Presented studies show brewer’s spent grains as a feasible enrichment for bricks’ raw material, allowing for the design of new products with greater strength, higher porosity and improved thermal isolation. Another way of the utilization of BSG is the production of biodegradable packages. Ferreira et al. created trays made out of BSG and starch, using the hot-pressing technique. The product was compared with typically expanded polystyrene trays. Results demonstrated that all of the prepared samples with BSG content from 40 to 80% represented higher flexural strength than non-biodegradable counterpart (from 2.62 to 1.51 MPa for BSG trays in comparison to 0.64 MPa for polystyrene). Unfortunately, flexural strength was decreased after contact with water. To overcome such a problem, the addition of chitosan and glyoxal was proposed. Trials showed that tested additions were effective and allowed for the production of a biodegradable counterpart for expanded polystyrene trays [115]. Moreover, BSG has been mentioned as a sustainable material that could be used for the manufacturing of electrodes [116].

## 7. Use of BSG in Agriculture

### 7.1. Animal Nutrition

BSG can be successfully used as feed additives in animal nutrition, mainly for cattle and pigs. The literature data also describe successful studies on the effect of feeding fish and poultry. Due to the specificity of their composition, they have not been used as feeds for horses, sheep, or goats [117,118].

Fresh brewer’s spent grain is characterized by high water content (70–85%) and easily fermenting components, such as non-sugar starch, pentosans, and pectin compounds. Protein content in dry matter is recorded at the level of about 20%, and fibre almost 60%. Therefore, it is considered for extremely perishable feed, and it can be used for a maximum of 2–3 days from manufacture provided that it is stored at 5 °C [117,119,120,121].

In practice, this means that they can only be used by farm buildings in the close vicinity of the brewery, for which the time and costs associated with obtaining the linings as a feed additive are profitable [119].

There are ways of preserving the raw BSG that allow prolonging its suitability for animal consumption. The choice of a specific method, such as pickling or drying, is primarily related to the costs of its use and the animal species that will be fed with BSG. The pickling of BSG has a positive effect on extending its shelf life, and it is most often used due to its low impact on the change of quality composition. Dried BSG is rarely used in animal nutrition, due to the relation between the cost of the drying process and the subsequent use of this feed [120,121].

Both pickled and untreated fresh BSG are used mainly in the feeding of dairy and fattening cattle. Milk-making properties characterize them. Hence, they are most often used in the initial and final stage of lactation. Due to their low fat and carbohydrate content, they should never be used as a complete independent feed, and they are most often served as an additive in combination with cereal shots, e.g., corn silage, green fodder, and protein-rich legumes. When applied in excessive quantities, they may cause diarrhea, decreased fertility of cows, and complications in the perinatal period [120,122].

The studies carried out on carp fish showed that, in experimental groups, the replacement of 10–40% of rice bran included in feed mixes with BSG resulted in improved body weight gain. According to the authors, this beneficial effect was also due to the content of high-quality protein with a good amino acid profile, especially cysteine, lysine, and methionine. Achieving an elevated content of these amino acids in brewer’s spent grain is possible thanks to the use of appropriately selected species of microorganisms involved in the production process [118]. Moreover, nutritional quality tests led by Nazzaro et al. showed that brewer’s by-products might be suitable for marine (*Sparus aurata*) and fresh (*Oncorhyncus mykiss*) fish nutrition, with digestibility up to 88% for both fish species [123]. Another approach for utilization of BSG is enzymatic pre-treatment of mentioned by-product in order to cleave remaining cellulose and protein chains [124].

In relation to poultry feeding, the use of BSG as feed has a significant impact on production because it increases the rate of hatching from fertilized eggs. Literature data, similarly as in the case of carp, also indicate a significant impact of the amino acids on the nutrition of laying hens, which, in turn, translates directly into other breeding indicators, thus increasing the quality of poultry production [118,120]. Additionally, some studies investigated the use of BSG as fodder for edible insects [125].

### 7.2. BSG as a Sustainable Fertilizer and Soil Amendment

Residues from the brewing industry contain a lot of valuable nutrients, such as phosphorus or potassium, which could be used as a source of nutrients for crops [126,127]. Spent grain (12.5 t/ha) was reported to be comparable with NPKF (200 kg/ha) and resulted in higher fruit yields when applied to soils in south-western Nigeria [128]. Moreover, some other trials reported a synergetic effect between the use of NPK and BSG on the growth of maize in the south-eastern part of Nigeria [129]. Some studies reported the effect of the synergy of BSG application, which could play a role of fertilizer and pest control for soil-borne insects, such as *G. mellonella* larvae [130].

Apart from the supply of nutrients, the application of BSG to the soil could be beneficial in terms of the improvement of the organic matter content [131]. This makes it an interesting choice for a feedstock for biochar production, with the intention of soil application.

Application of biochar to soil offers important benefits, including more efficient use of nutrients, improved soil quality, as well as increased water holding capacity [132,133,134,135]. Moreover, it promotes an increase in the diversity of soil microbial community by changing the root-associated microbiome [136,137,138,139,140,141]. Furthermore, it allows the soil to effectively become a carbon sink [142]. Reported soil application of hydrochars from spent brewer’s yeast resulted in a positive effect on the soil aggregation [143]. Application of BSG to the cultivation of hops resulted in significant improvements in the growth of the root system of the plants [144].

## 8. Human Nutrition

**Figure 3:**
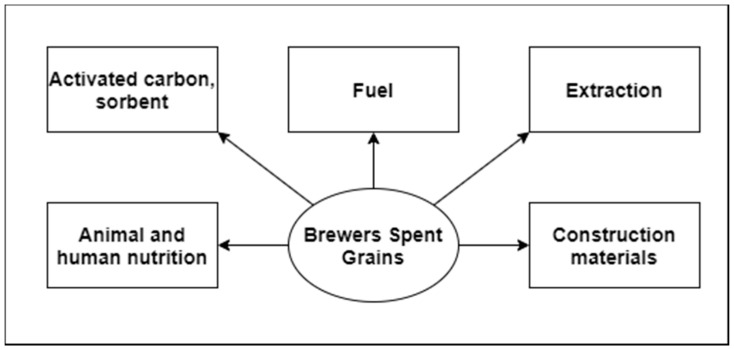
Possible ways to utilize BSG.

One of the most popular methods of utilization of the by-product of beer production, which is BSG, is to use it as animal feed or (often after some modifications) as an addition to human food (Figure 3.). BSG contains many desirable elements of the human diet, such as vitamins, fibre, or minerals [59,145]. However, due to the high moisture content [18], the linings must be treated freshly after beer processing (e.g., dried or frozen) to avoid the multiplication of microorganisms on them. Brewer’s spent grain can be used for, among other things, the following purposes:

### 8.1. Beer Production

Due to the remaining sugars, which have not passed into the wort as a result of the mashing or lautering process (e.g., during the brewing of high extract beers), BSG is also suitable for beer production. Studies show that the first 5% of BSG from the top layer after filtration may contain undigested starch [146]. They may be used as an addition for the production of the next beer, replacing part of the backfill. Additionally, when the mashing regime is changed, especially during the production of low-alcoholic or non-alcoholic beers, the starch present in BSG is not fully digested and leached into wort [147]. Again, these BSG could be used in the production of beer with lower alcohol content.

### 8.2. Flour, Pasta and Bread Production

In recent years, there have been attempts to enrich flour with various additions like split pea or bean [148]. Nocente et al. showed that enrichment of durum flour with BSG increases fibre up to 135%, β-glucan up to 85% and total antioxidant capacity up to 19% in comparison with all wheat durum flour. Authors claim that 10% addition of BSG is optimal in terms of organoleptic and technological properties of BSG-enriched pasta [149]. The addition of flour made of BSG has increased water absorption by the bread and may have a positive effect on its texture and volume compared to bread made of standard flour [150]. With a higher content of flour made of BSG in baked goods, they are characterized by a higher content of fibre [151]. However, they disturb the dough forming and contribute to lower gas retention and, consequently, lower volume of baked goods. This effect can be eliminated by adding enzymes such as xylanase and lipase during bread baking [152,153]. The addition of LE, PE, and PCE also positively influences the volume of the loaf, its ageing rate, and crumb structure. Studies recommend not exceeding the value of 30% of the ratio of BSG flour to total flour volume in the case of bread baking [151].

### 8.3. Cookies

As in the case of bread, the addition of flour from BSG affects the qualities of cookies such as appearance, hardness, chewiness, smell, and taste. Researchers testing cookies with different content of flour from BSG rated them on a scale from 1 to 5, all with BSG flour between 3.5 and 4.5 (where 4 means “like moderately”). However, they fall out worse compared to cookies made of wheat flour only, which were rated 5 (“like very much”) [154]. Moreover, the addition of BSG flour to wheat flour in ratio 1:4 may decrease the glycemic index of cookies [155]. A similar ratio of wheat flour to BSG flour (3:1), according to the researchers, had the best taste qualities among the cookies from the research trials [154]. Furthermore, cookies made of brewer’s spent grains are quite popular among homebrewers [156,157,158].

### 8.4. Snacks

Snacks with BSG contain a large amount of fibre and protein. However, the content of a large amount of water-insoluble fibre—lignin and cellulose increase the hardness of snacks, which directly causes worse taste qualities. This effect can be mitigated by adding corn starch and whey protein isolate [155]. In the case of crispy-slices production, the content of 10% of flour from BSG did not affect the taste and consistency of crispy-slices and contributed to an increase in the fibre content in the produced snack [159]. Stojceska et al. reported that BSG might be added to snack extrudates up to 20% in order to obtain product similar to commercially available snacks, although 30% addition still ensures acceptable physicochemical properties [160].

### 8.5. Frankfurters

Another possible way to implement BSG into the food industry is meat production. Özvural et al. enriched frankfurters with brewer’s spent grains. Although the addition of BSG reduced sensory impressions compared to the control group without BSG (7.57 on a 9-point scale, where 1 = dislike extremely and 9 = like extremely), they were still at an acceptable level (from 5.47 to 7.13). The study shows that BSG has a potential in the production of meat products with increased fibre and reduced fat content [161]. What is more, such an approach may reduce the costs of the final product.

### 8.6. High Fibre Products and High Protein Products

Researchers from Virginia Tech suggested obtaining high fibre product (HFP) and high protein product (HPP) by wet fractionation process [9]. From the tested reagents (i.e., sodium hydroxide, sodium bisulfite, and alcalase), the best effects were observed for alcalase. Under optimal conditions, HPP with a recovery rate of 43.7% and protein content of 42.8% *w*/*w* was obtained. The dominant amino acids in HPP composition were glutamic acid (20.8% *w*/*w*), proline (7.5% *w*/*w*), and leucine (10.5% *w*/*w*). The produced HFP had more than 80% of fibre, consisting of hemicellulose (about 42% *w*/*w*), cellulose (about 24% *w*/*w*), and lignin (about 10% *w*/*w*). In case of a positive analysis of HPP and HFP production costs, there is another possibility of commercial application of BSG in the development of the agriculture and food industry.

## 9. Conclusions

Brewer’s spent grains are standardized and rich biowastes. They are being produced in large breweries daily, but due to its high moisture content, its transportation is costly. The best way to utilize the mentioned by-product is processing in the neighborhood of the breweries in order to reduce the costs of transport. On the other hand, drying of BSG using waste heat from breweries may be a feasible way to produce desirable raw material for other branches of industry. That may simplify waste management in breweries or even provide additional income for large brewing facilities. In the case of restaurant microbreweries, BSG may be a chance to enrich their gastronomic offer with healthy dishes. Further studies may show a new application of brewer’s solid wastes. Moreover, due to the circular economy trend of upcycling agro-food wastes, BSG is likely to be used in numerous branches of industry as well as in agriculture.

## Figures and Tables

**Table 1:** The approximate chemical composition of BSG in different studies (% of dry weight).

	Lignin	Cellulose	Hemicellulose	Ash	Protein	Lipids	Phenolics	Starch
Kanauchi et al., (2001) [13]	11.9	25.4	21.8	2.4	24.0	10.6	N.D.	N.D.
Carvalheiro et al., (2004) [14]	21.7	21.9	29.6	1.2	24.6	N.D.	N.D.	N.D.
Silva et al., (2004) [15]	16.9	25.3	41.9	4.6	N.D.	N.D.	N.D.	N.D.
Mussatto and Roberto, (2006) [16]	27.8	16.8	28.4	4.6	15.2	N.D.	N.D.	N.D.
Celus et al., (2006) [17]	N.D.	0.3	22.5	3.3	26.7	N.D.	N.D.	1
Xiros et al., (2008) [18]	11.5	12	40	3.3	14.2	13	2.0	2.7
Jay et al., (2008) [19]	20–22	31–33	N.D.	N.D.	15–17	6–8	1.0–1.5	10–12
Treimo et al., (2009) [20]	12.6 ± 0.1	45.9 *			23.4 ± 1.4	N.D.	N.D.	7.8 ± 0.2
Robertson et al., (2010) [21]	13–17	N.D.	22–29	N.D.	20–24	N.D.	N.D.	2–8
Khidzir et al., (2010) [22]	56.74 ± 9.38	40.20 ± 17.71	N.D.	2.27 ± 0.76	6.41 ± 0.31	2.50 ± 0.11	N.D.	0.28 ± 0.06
Waters et al., (2012) [23]	N.D.	26.0	22.2	1.1	22.1	N.D.	N.D.	N.D.
Nuno et al., (2013) [24]	19.40 ± 0.34	21.73 ± 1.36	19.27 ± 1.18	4.18 ± 0.03	24.69 ± 1.04	N.D.	N.D.	N.D.
Sobukola et al., (2012) [25]	9.19 ± 0.011	60.64 ± 0.26 *		2.48 ± 0.02	24.39 ± 0.46	6.18 ± 0.13	N.D.	N.D.
Kemppai-nen et al., (2016) [26]	19.6	45 *		4.1	20.3	N.D.	N.D.	N.D.
Yu et al., (2020) [27]	N.D.	51.0 ± 0.7 *		4.1 ± 0.1	23.4 ± 0.2	9.4 ± 0.1	N.D.	N.D.

N.D.—no data, *—all carbohydrates.
